# Prevalence and Risk Factors of Choledocholithiasis in Omani Patients With Sickle Cell Disease Undergoing Endoscopic Retrograde Cholangiopancreatography: A Retrospective Analysis

**DOI:** 10.7759/cureus.51133

**Published:** 2023-12-26

**Authors:** Khalid Al Shamousi, Maimoona Al Maimani

**Affiliations:** 1 Medicine, Sultan Qaboos University, Muscat, OMN; 2 Internal Medicine, College of Medicine and Health Sciences, Sultan Qaboos University, Muscat, OMN

**Keywords:** endoscopic retrograde cholangiopancreatography, sultan qaboos university hospital, oman, biliary stones, cholecystolithiasis, choledocholithiasis, sickle cell disease

## Abstract

Background

Sickle cell disease (SCD) is a prevalent genetic disorder in the Middle East, particularly in Oman, leading to significant morbidity. It is caused by a mutation in the gene encoding hemoglobin (Hb) molecules, resulting in the formation and polymerization of hemoglobin S (HbS), which subsequently leads to hemolysis. Chronic hemolysis in SCD patients often results in various complications, including increased bilirubin levels in the gallbladder and the formation of pigmented biliary stones, which may obstruct the biliary tract system. In such cases, endoscopic retrograde cholangiopancreatography (ERCP) is often employed as a diagnostic and therapeutic tool to manage biliary complications.

Objectives and rationale

Considering the lack of studies on the Omani population with SCD, our study aims to determine the incidence of biliary stone formation in SCD patients undergoing ERCP and identify associated risk factors.

Subjects and methods

This retrospective study included 79 SCD patients aged over 12 years who underwent ERCP at Sultan Qaboos University Hospital, Muscat, Oman, between January 2010 and January 2023. Patient data were extracted from medical records. Continuous variables were analyzed using mean and standard deviation calculations, with independent sample t-tests for mean comparisons. The chi-square test assessed associations between categorized variables, with a p-value of ≤0.05 denoting statistical significance.

Results

The prevalence of choledocholithiasis in SCD patients undergoing ERCP was 67.1%. The incidence was higher in females (68.9%) than males (65.9%), in patients aged 12-29 (71.2%) compared to those aged ≥29 (59.3%), in patients with SCD (70.6%) versus sickle cell thalassemia (66.1%), and in those who had undergone gallbladder removal (80.0%) compared to those who did not (61.3%). Prevalence was also higher in patients not using hydroxyurea and folic acid (70.6% and 84.6%, respectively); however, chi-square analysis showed no significant association (p-value > 0.05). Additionally, t-test comparisons of HbS and HbF levels showed no significant differences.

Conclusion

This study documents a high prevalence of choledocholithiasis (67.1%) in SCD patients undergoing ERCP. Although the prevalence is notable, the examined risk factors did not show a significant association with stone formation.

## Introduction

Sickle cell disease (SCD) is a group of inherited genetic disorders resulting from mutations in the genes that encode for the hemoglobin (Hb) β subunit [1]. Hb, a tetrameric protein molecule found in red blood cells (RBCs), functions as an oxygen carrier. This Hb molecule is encoded by different globin genes at various life stages; for instance, fetal Hb (HbF) consists of two α globin subunits and two ϒ globin subunits, whereas HbA, the most abundant type in adults, is made of two α globin subunits and two β globin subunits [1].

Pathogenesis of SCD involves a mutation at the sixth position of the DNA coding for the β globin subunit's amino acids, changing from adenine to thymine. This mutation alters the amino acid from glutamic acid to valine, forming the sickled Hb molecule (HbS) [2]. Oxygen tension significantly affects the function of HbS; at high oxygen tension, Hb remains a monomer in oxygenated RBCs. However, under low oxygen tension, deoxygenated RBCs cause HbS to become insoluble and start polymerizing, cross-linking with other HbS molecules inside the RBCs and forming crystals. This process changes the RBCs into a sickle shape, characteristic of SCD [1,2].

Different forms of SCD exist, each characterized by the presence of HbS instead of normal HbA. The most common type, sickle cell anemia (HbS/S), is a homozygous HbS disorder manifesting when a person inherits two alleles of the sickle mutation in the β globin gene. SCD can also be manifested by inheriting one allele of βS with other β globin gene variants, resulting in heterozygous variants such as HbS/C, HbS/O, HbS/D, and HbS/E diseases. Additionally, βS can coexist with the β thalassemia allele, leading to HbS/β-thalassemia [1,3].

Complications of SCD include polymerization of HbS in RBCs after low oxygen tension, leading to sickle-shaped cells that lose flexibility, become more rigid, and are prone to destruction. These changes in the RBCs result in a shorter lifespan, vessel occlusion, disrupted blood flow, vaso-occlusive crises, and hemolytic crises [1,4,5]. Hemolysis releases Hb, which is degraded into unconjugated bilirubin, which metabolizes in the liver into conjugated bilirubin [6,7]. Excessive lysis in SCD increases unconjugated bilirubin levels, leading to bilirubin saturation in bile and the formation of biliary stones [7]. These stones are classified as intrahepatic (hepatolithiasis) or extrahepatic (choledocholithiasis and cholecystolithiasis) [8].

Chronic hemolysis in patients with SCD is a known risk factor for choledocholithiasis [9-11]. However, there are other risk factors in these patients, such as dehydration and sickle cell cholangiopathy [4,5]. This study aims to define the association of risk factors with choledocholithiasis in SCD patients, as no previous studies have been conducted in Oman, where approximately 3.8% of the population has SCD [12].

## Materials and methods

Study design and setting

This single-center, retrospective cross-sectional study was conducted at Sultan Qaboos University Hospital (SQUH), Muscat, Oman. The study's design aimed to analyze existing electronic patient records to identify prevalence, patterns, and outcomes in SCD patients undergoing endoscopic retrograde cholangiopancreatography (ERCP).

Study participants and duration

A total of 79 patients with SCD who underwent ERCP at SQUH were included in the study. The participants were observed over a 13-year period, from January 2010 to January 2023, allowing for a comprehensive evaluation of long-term outcomes.

Inclusion and exclusion criteria

The study focused on patients diagnosed with SCD who underwent ERCP in the study period and were aged over 12 years. These criteria were chosen to ensure that the study focused on a relevant and specific patient population. Patients who did not meet these criteria were excluded from the study.

Data collection

Data were collected using the Hospital Information System (Trackcare) at SQUH. This included demographic data (governorate, gender, age at ERCP), clinical data (type of SCD, ERCP findings of choledocholithiasis), surgical history (gallbladder and spleen removal), medication history (use of hydroxyurea and folic acid), and Hb variants (HbS, HbF).

Statistical analysis

Data analysis was conducted using SPSS Statistics software, Version 25 (IBM Corp., Armonk, NY). A pie chart was used to illustrate the prevalence of biliary stones among the patient cohort. The chi-square test was used to assess associations between categorized variables, and an independent sample t-test was used to compare means between groups. Statistical significance was established at a p-value of ≤0.05.

Ethical considerations

Ethical approval for the study was obtained from the Medical Research Ethics Committee (MREC) at the College of Medicine and Health Sciences, Sultan Qaboos University (Approval No. MREC # 2817), in compliance with ethical research guidelines. Due to the retrospective nature of the study, patient consent was not required, and ethical clearance was instead sought from the MREC.

## Results

**Figure 1:**
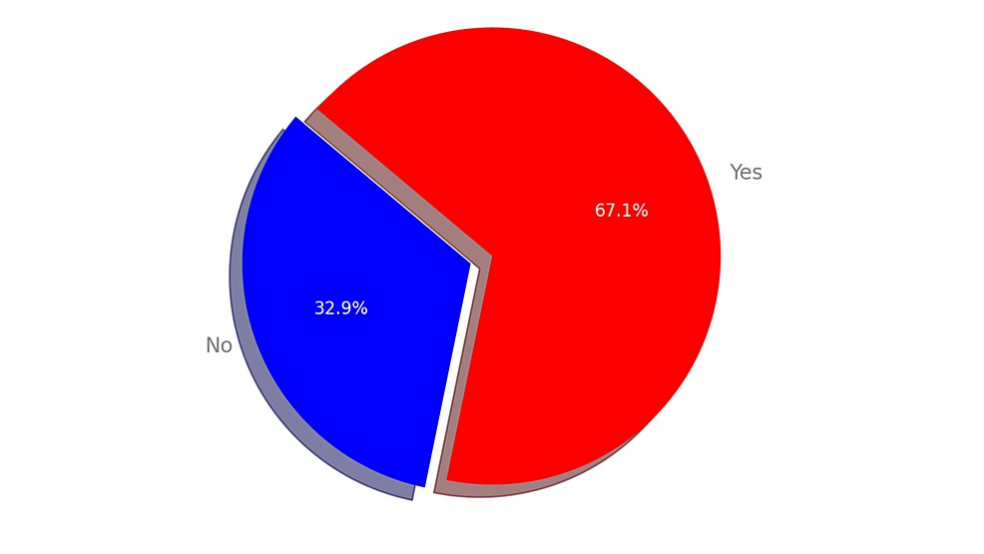
Prevalence of choledocholithiasis in 79 SCD patients undergoing ERCP. SCD, sickle cell disease; ERCP, endoscopic retrograde cholangiopancreatography

During the period of January 2010 to January 2023, a total of 79 patients with SCD underwent ERCP. Out of these, only 53 (67.1%) patients were diagnosed with choledocholithiasis during the procedure, as shown in Figure 1.

Demographics

**Figure 2:**
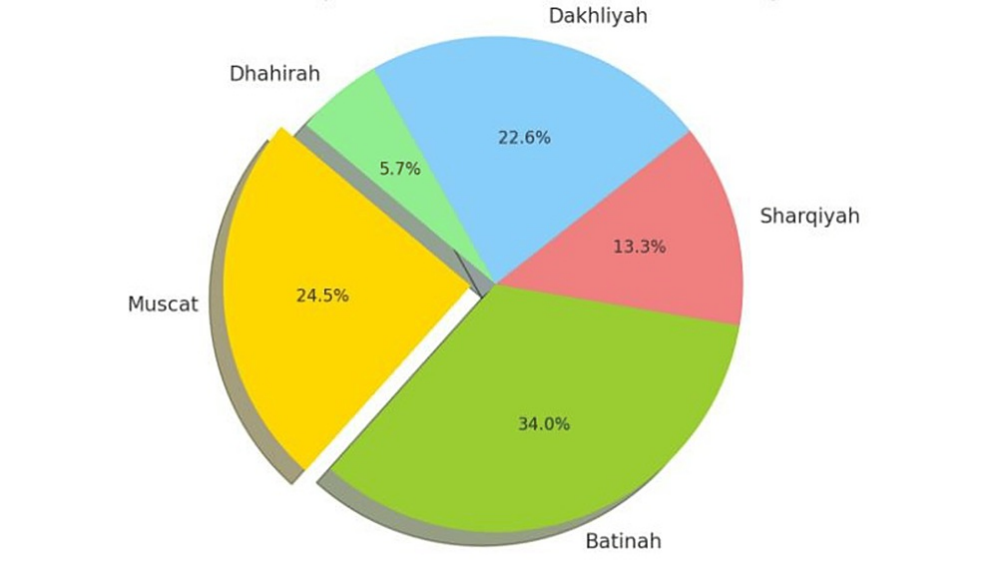
Prevalence of choledocholithiasis in 79 SCD patients undergoing ERCP across five governorates in Oman. SCD, sickle cell disease; ERCP, endoscopic retrograde cholangiopancreatography

The pie chart in Figure 2 illustrates that most patients with SCD who developed choledocholithiasis were from Al Batinah (34.0%). Nearly a quarter of the patients were from Muscat and A’Dakhiliyah (24.5% and 22.6%, respectively). In addition, 13.2% were from A’Sharqiyah. Only 5.7% were from A’Dhahirah, which represents the minority of patients.

As shown in Table 1, 65.9% of patients who developed choledocholithiasis were males, compared to 68.9% of females. Furthermore, choledocholithiasis was found to be high in patients aged 12 to 29 years. Around 71.2% of patients aged 12-29 years developed choledocholithiasis, while 59.3% aged more than 29 years had choledocholithiasis. However, none of these variables were statistically significant because the p-value was >0.05 (0.993 for gender and 0.415 for age).

**Table 1:** Distribution of demographic data of patients with SCD who underwent ERCP regarding the presence or absence of choledocholithiasis. *Based on the chi-square test SCD, sickle cell disease; ERCP, endoscopic retrograde cholangiopancreatography

Factors		Choledocholithiasis				X^2^	P-value^*^
		Yes		No			
		n	%	n	%		
Age	12-29 years	37	71.2%	15	28.8%	1.139	0.415
	≥29 years	16	59.3%	11	40.7%		
Gender	Male	29	65.9%	15	34.1%	0.063	0.993
	Female	24	68.9%	11	31.4%		

Clinical features

The frequency of choledocholithiasis was higher among patients with sickle cell anemia than sickle cell thalassemia. As indicated in Table 2, 70.6% of patients with sickle cell anemia developed choledocholithiasis in contrast with 66.1% of patients with sickle cell thalassemia. Even so, this was statistically non-significant, with a p-value of 0.956. Moreover, 80.0% of patients had their gallbladder removed (cholecystectomy) after developing choledocholithiasis, while 61.3% did not, and 55.6% removed it before they had stones. However, this was non-significant (p-value = 0.138). Over and above that, 66.6% of patients had stones after spleen removal (splenectomy), whereas 67.3% had stones with an intact spleen. This percentage showed no significant association between spleen removal and choledocholithiasis in patients with SCD (p-value = 1.000), as illustrated in Table 2.

**Table 2:** Distribution of clinical data of patients with SCD who underwent ERCP regarding the presence or absence of choledocholithiasis. *Based on the chi-square test SCD, sickle cell disease; ERCP, endoscopic retrograde cholangiopancreatography

Factors		Choledocholithiasis				X^2^	P-value^*^
		Yes		No			
		n	%	n	%		
Type of SCD	Sickle cell anemia	12	70.6%	5	29.4%	0.120	0.956
	Sickle cell thalassemia	41	66.1%	21	33.9%		
Gallbladder removal (cholecystectomy)	Yes, after ERCP	24	80.0%	6	20.0%	3.821	0.138
	Yes, Before ERCP	10	55.6%	8	44.4%		
	no	19	61.3%	12	38.7%		
Spleen removal (splenectomy)	yes	18	66.6%	9	33.3%	0.003	1.000
	no	35	67.3%	17	32.7%		

Drug history

As shown in Table 3, 64.4% of patients who used hydroxyurea and 63.6% who used folic acid had choledocholithiasis. In comparison, 70.6% and 84.6% who did not use hydroxyurea and folic acid, respectively, had stones, although this was not significant (p-value = 0.739 for using hydroxyurea and 0.123 for using folic acid).

**Table 3:** Distribution of past drug history of patients with SCD who underwent ERCP regarding the presence or absence of choledocholithiasis. *Based on the chi-square test SCD, sickle cell disease; ERCP, endoscopic retrograde cholangiopancreatography

Factors		Choledocholithiasis				X^2^	P-value^*^
		Yes		No			
		n	%	n	%		
Hydroxyurea	Yes	29	64.4%	16	35.6%	0.331	0.739
	No	24	70.6%	10	29.4%		
Folic acid	Yes	42	63.6%	24	36.4%	2.165	0.123
	No	11	84.6%	2	15.4%		

Laboratory investigations

HbS and HbF concentrations were higher among patients who develop choledocholithiasis (65.84±20.89 and 8.24±5.74, respectively) than patients who did not (63.50±18.38 and 7.42±5.43, respectively). However, no significant association was observed between them (p-value = 0.628 for HbS and 0.544 for HbF), as shown in Table 4.

**Table 4:** Distribution of hemoglobin amount in patients with SCD who underwent ERCP regarding the presence or absence of choledocholithiasis. *Based on the chi-square test SCD: sickle cell disease; ERCP, endoscopic retrograde cholangiopancreatography

Factors	Choledocholithiasis		t	P-value^*^
	Yes	No		
	Mean ±SD	Mean ±SD		
Hemoglobin S	65.84 ±20.89	63.50 ±18.38	0.487	0.628
Hemoglobin F	8.24 ±5.74	7.42 ±5.43	0.609	0.544

## Discussion

Our study's findings indicate a higher prevalence (67.1%) of choledocholithiasis in SCD patients undergoing ERCP compared to previous studies. For instance, a small study at Duke University Medical Center with a sample size of 35 reported a different prevalence rate (26%) [13]. This variation could be attributed to the discrepancy in study sizes and potential genetic differences in the populations examined. Additionally, a Saudi Arabian study from 1988 to 1996, including 52 children, reported that 46% of SCD patients had biliary stones [14], similar to a Brazilian study that found a 45% prevalence [9]. Our study's strict inclusion criteria, focusing solely on patients undergoing ERCP, may have contributed to our higher prevalence rate.

We found no significant association between stone development and gender, which aligns with the findings of a study conducted in Brazil [4]. Furthermore, a study in Saudi Arabia on patients aged 2 to 18 years also showed no significant association between stones and gender [10]. In terms of age, our study found that 71.4% of patients with SCD who developed stones were aged 12 to 29 years, which was higher than those aged more than 29 years. However, this did not represent a statistically significant association (p-value = 0.415), contrary to the results of a Brazilian study from 1995 to 2014, which reported a significant association in this age group (p-value = 0.018) [4]. A possible explanation for this will be the increasing hemolysis during intense physical activity, which is variable among different age groups [15].

In terms of SCD type, patients with sickle cell anemia in our study had a higher percentage of stone development compared to those with sickle cell thalassemia. Yet, no significant difference was observed, which can be attributed to the fact that any chronic hemolytic disease can increase the formation of pigmented stones. This observation was consistent with previous research conducted in Brazil and Saudi Arabia [4,10]. Gumiero et al. reported a statistically significant predominance of 63.5% in patients with sickle cell anemia [9]. In contrast, our study aimed to look for any association between the two variables, finding that 80.0% of patients who underwent cholecystectomy due to stones showed no significant findings. Interestingly, we found no association between the 66.6% of patients who underwent splenectomy and choledocholithiasis, indicating a need for more research to study this relationship, as no substantial data were found except for a study in Qatif Central Hospital, Saudi Arabia, which reported that 24% of patients with SCD who had stones had undergone a splenectomy [14].

Regarding treatment, we found no statistical association of the use of folic acid and hydroxyurea with choledocholithiasis (p-value = 0.123 and 0.739, respectively), in agreement with the findings of Martins et al. and Alhawsawi et al. [4,10]. Given the retrospective nature of our study, we could not confirm the adherence to these medications, which are expected to reduce the amount of hemolysis and thus reduce the risk of pigmented stones physiologically [16].

Contrary to expectations, our findings revealed no significant correlation between the levels of HbS in the blood and the development of choledocholithiasis. This result is in contrast to the findings reported by Alhawsawi et al., who identified a significant association between HbS levels and stone development (p-value = 0.041) [10]. This discrepancy could be due to variations in the patient cohorts, methodology, or other unidentified factors influencing stone formation in SCD patients. Furthermore, our study did not find a significant association between levels of fetal hemoglobin (HbF) and choledocholithiasis. This finding was consistent with results from a study conducted in Saudi Arabia [14], which also did not observe a significant relationship between HbF levels and the development of biliary stones in SCD patients. Given these findings, we believe that further research is warranted, particularly focusing on genetic variations that might exist between populations, such as between Omani and Saudi patients. Such studies could provide deeper insights into the pathophysiological mechanisms of choledocholithiasis in SCD and the role of various types of Hb in this process.

The study's limitations, including its retrospective design, single-center scope, and relatively small sample size, provide a roadmap for future research endeavors. These findings advocate for larger-scale, multicenter studies across Oman to capture a more representative sample and to better understand the geographical variations in disease prevalence, particularly the notable rate of biliary stone development in the Al Batinah governorate.

## Conclusions

Our comprehensive retrospective study at SQUH provides crucial insights into the high prevalence of choledocholithiasis among SCD patients undergoing ERCP, with an observed rate of 67.1%. This finding is significant as it underscores the need for heightened clinical vigilance and potential preventive strategies for this patient population. Despite the rigorous analysis, our study did not establish a statistically significant correlation between the evaluated risk factors and the development of choledocholithiasis, presenting an intriguing contrast to existing literature. This highlights the possibility of unique regional or genetic factors influencing disease manifestation and progression, warranting further in-depth investigation.

The implications of our research are far-reaching, suggesting potential revisions to screening and management protocols for SCD patients, especially in areas with a high disease prevalence. As we continue to unravel the complexities of SCD and its complications, our ultimate goal remains to enhance patient outcomes through evidence-based medicine, early intervention, and personalized care strategies.
